# Unique properties of a *Dictyostelium discoideum* carbohydrate-binding module expand our understanding of CBM–ligand interactions

**DOI:** 10.1016/j.jbc.2022.101891

**Published:** 2022-04-01

**Authors:** Marcelo Vizona Liberato, Bruna Medeia Campos, Geizecler Tomazetto, Lucy Isobel Crouch, Wanius Garcia, Ana Carolina de Mattos Zeri, David Nichol Bolam, Fabio Marcio Squina

**Affiliations:** 1 Programa de Processos Tecnológicos e Ambientais, Universidade de Sorocaba (UNISO), Sorocaba, SP, Brazil; 2 Laboratório Nacional de Biociências (LNBio), Centro Nacional de Pesquisa em Energia e Materiais (CNPEM), Campinas, São Paulo, Brazil; 3 Department of Biological and Chemical Engineering (BCE), Aarhus University, Aarhus, Denmark; 4 Institute of Microbiology and Infection, College of Medical and Dental Sciences, University of Birmingham, Birmingham, United Kingdom; 5 Centro de Ciências Naturais e Humanas, Universidade Federal do ABC (UFABC), Santo André, São Paulo, Brazil; 6 Laboratório Nacional de Luz Sincrotron (LNLS), Centro Nacional de Pesquisa em Energia e Materiais (CNPEM), Campinas, São Paulo, Brazil; 7 Institute for Cell and Molecular Biosciences, The Medical School, Newcastle University, Newcastle, United Kingdom

**Keywords:** carbohydrate-binding protein, X-ray crystallography, ligand specificity, cellulose, CAZymes, AGE, affinity gel electrophoresis, BMCC, bacterial microcrystalline cellulose, CAZy, carbohydrate-active enzymes database, CBM, carbohydrate-binding module, GH, glycoside hydrolase, HEC, hydroxyethyl cellulose, MW, molecular weight, PDB, Protein Data Bank

## Abstract

Deciphering how enzymes interact, modify, and recognize carbohydrates has long been a topic of interest in academic, pharmaceutical, and industrial research. Carbohydrate-binding modules (CBMs) are noncatalytic globular protein domains attached to carbohydrate-active enzymes that strengthen enzyme affinity to substrates and increase enzymatic efficiency *via* targeting and proximity effects. CBMs are considered auspicious for various biotechnological purposes in textile, food, and feed industries, representing valuable tools in basic science research and biomedicine. Here, we present the first crystallographic structure of a CBM8 family member (CBM8), DdCBM8, from the slime mold *Dictyostelium discoideum*, which was identified attached to an endo-β-1,4-glucanase (glycoside hydrolase family 9). We show that the planar carbohydrate-binding site of DdCBM8, composed of aromatic residues, is similar to type A CBMs that are specific for crystalline (multichain) polysaccharides. Accordingly, pull-down assays indicated that DdCBM8 was able to bind insoluble forms of cellulose. However, affinity gel electrophoresis demonstrated that DdCBM8 also bound to soluble (single chain) polysaccharides, especially glucomannan, similar to type B CBMs, although it had no apparent affinity for oligosaccharides. Therefore, the structural characteristics and broad specificity of DdCBM8 represent exceptions to the canonical CBM classification. In addition, mutational analysis identified specific amino acid residues involved in ligand recognition, which are conserved throughout the CBM8 family. This advancement in the structural and functional characterization of CBMs contributes to our understanding of carbohydrate-active enzymes and protein–carbohydrate interactions, pushing forward protein engineering strategies and enhancing the potential biotechnological applications of glycoside hydrolase accessory modules.

Glycoside hydrolases (GHs) are enzymes capable of breaking glycosidic bonds. They are found in all living beings and are involved in essential functions, such as cell wall modeling, defense, symbiosis, signaling, biosynthesis, and nutrient acquisition (1). These enzymes are broadly applied in the industrial production of paper, fabrics, and food and have gained visibility in biorefining processes for conversion of biomass into renewable fuels and chemicals (2, 3, 4).

GHs are composed of a catalytic domain, which is often covalently linked to one or more accessory modules that regulate their activity, such as carbohydrate-binding modules (CBMs). CBMs are discrete folding units capable of binding to different types of carbohydrates, and their main role is to mediate the interaction between the enzyme and the target substrate, leading to modifications in catalytic efficiency (5). More specifically, CBMs perform functions such as increasing substrate accessibility through disruption of the crystalline structure of cellulose (6, 7), promoting specificity (8), and complementing the substrate-binding site of catalytic domains (9). To date, CBMs have been grouped into 89 different families in the carbohydrate-active enzymes (CAZy) database based on amino acid sequence similarities (10). CBMs are also classified based on their functional properties: type A CBMs possess flat binding faces, capable of binding to crystalline polysaccharides; type B CBMs bind internally to soluble polysaccharides *via* cleft-shaped contact sites; and type C CBMs interact with the terminal regions of carbohydrates through protein pocket–shaped sites (5, 11).

CBMs are considered auspicious for various biotechnological purposes, such as modification of the physical properties of fibers and potentiating enzymatic degradation of polysaccharides (12, 13), suitable for application in textile, food, and feed industries. CBMs are valuable tools in basic science research, used on *in situ* visualizations of polysaccharides, *in vivo* expression in plant physiology studies (14), and high-throughput analysis of polysaccharides based on microarrays (15). In synthetic biology initiatives, these protein modules are used as building blocks to construct minicellulosomes (16). In biomedicine, CBMs are employed to functionalize carbohydrate-based biomaterial and improve recombinant protein technology, promoting expression, purification, stabilization, and immobilization of heterologous proteins (17, 18, 19). In this sense, the CBMs were employed for expression at the surface of proteins of pathogens (17), such as antigenic protein fragments of severe acute respiratory syndrome coronavirus 2 (20).

The number of different sequences deposited in CAZy and the number of families have been rapidly increasing because of next-generation sequencing technologies (21). Occasionally, members from novel or poorly studied families reveal new characteristics that show uncertainties in the current classifications (22) as well as disclose opportunities to be explored for biotechnological purposes. Therefore, we selected CBM8 (DdCBM8) from the endo-β-1,4-glucanase (CelA) of *Dictyostelium discoideum* (Fig. 1). DdCBM8 is located at the C-terminal region of CelA and is connected to the catalytic GH9 domain through a threonine–glutamate–threonine–proline repeat linker (23). According to our studies, the DdCBM8 presented a planar binding site and has the ability of binding to insoluble crystalline cellulose, conferring type A CBM characteristics. However, DdCBM8 showed highest affinity for soluble polysaccharides, such as glucomannan, resembling type B CBMs. We aimed to study the DdCBM8 using biochemical and biophysical methods to determine the unique structural and functional properties to provide novel insights into CBM–ligand interactions that can be useful for protein engineering strategies, increasing the biotechnological application space of GH accessory modules.

**Figure 1:**
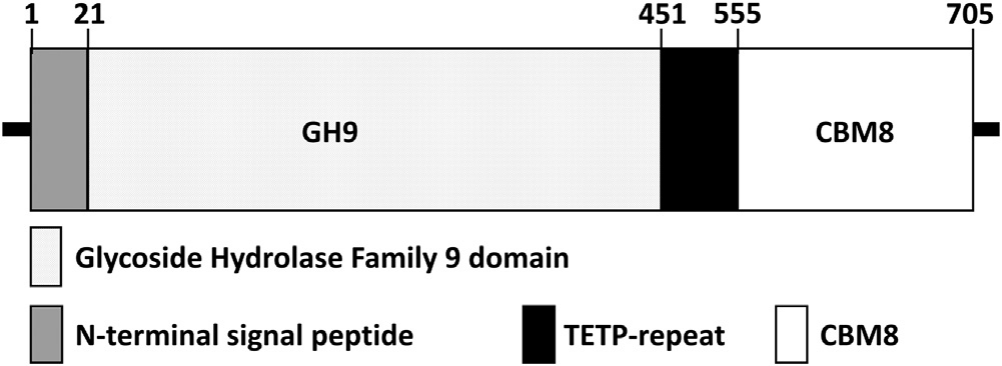
**Schematic representation of *Dictyostelium discoideum* Cel9A primary structure (UniProt ID:****P22699****).**

## Results

### Ligand-binding properties of DdCBM8

The gene encoding DdCBM8, comprising amino acid residues 555 to 705 from full-length CelA (Fig. 1), was inserted into the pET28a(+) bacterial vector, expressed in soluble form using *Escherichia coli* as the host, and purified to electrophoretic homogeneity using affinity and size-exclusion chromatography (data not shown).

**Figure 2:**
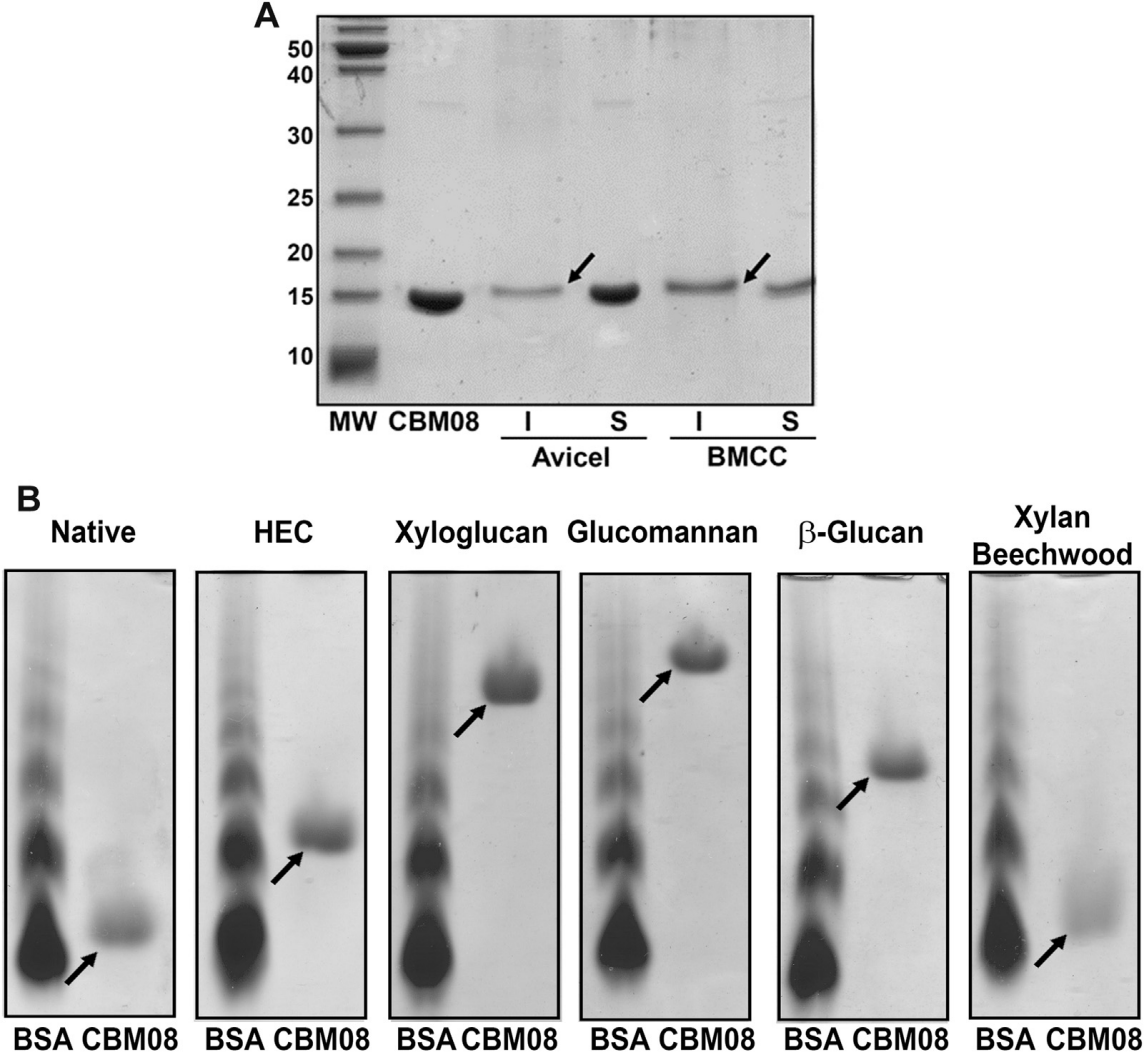
**Qualitative assessment of binding of DdCBM8 to soluble and insoluble polysaccharides.***A*, pull-down assay with insoluble forms of cellulose (Avicel and BMCC). The *arrows* indicate the protein bound to Avicel or BMCC (insoluble fraction). *B*, affinity gel electrophoresis. The experiment demonstrated that DdCBM8 binds to β-glucan, glucomannan, xyloglucan, and HEC but not to xylan. The *arrows* indicate the band corresponding to DdCBM8. BSA was used as a control as it does not interact with carbohydrates. BMCC, bacterial microcrystalline cellulose; BSA, bovine serum albumin; CBM, carbohydrate-binding module; DdCBM8, purified DdCBM8; HEC, hydroxyethyl cellulose; I_Avicel_, insoluble fraction from the assay with Avicel; I_BMCC_ = Insoluble fraction from the assay with BMCC; MW, molecular weight marker (PageRuler Unstained Protein Ladder); S_Avicel_, soluble fraction from the assay with Avicel; S_BMCC_, soluble fraction from the assay with BMCC.

**Figure 3:**
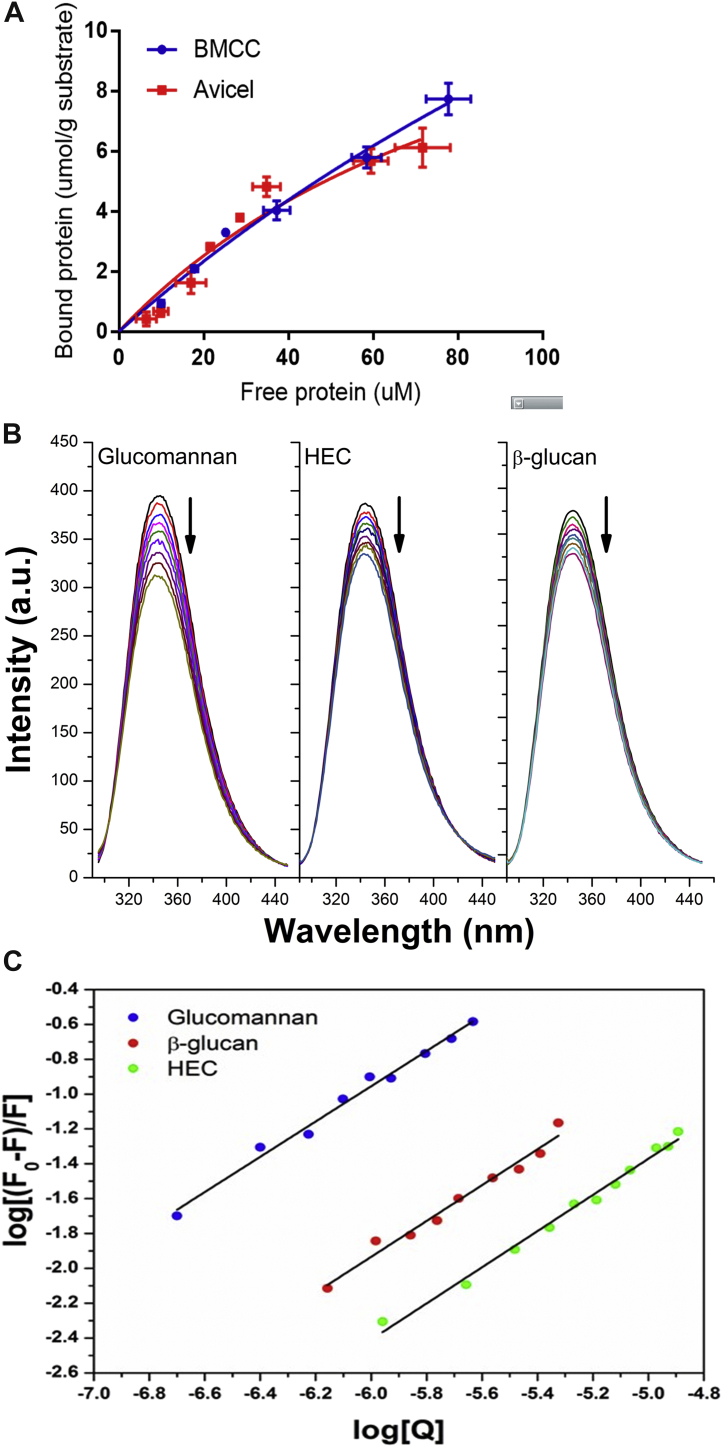
**Quantitative assessment of binding of DdCBM8 to soluble and insoluble polysaccharides.***A*, depletion isotherms of DdCBM8 binding to insoluble forms of cellulose (Avicel and BMCC). *B*, intrinsic fluorescence spectroscopy of DdCBM08 in the absence and presence of different soluble polysaccharide concentrations (glucomannan, HEC, and β-glucan). The *arrows* indicate the reduction of fluorescence intensity caused by addition of increasing concentration of polysaccharide. *C*, double logarithmic plot of log ([*F*_0_–*F*]/*F*]) *versus* log [Q] derived from the fluorescence quenching of DdCBM8 induced by soluble polysaccharides (Q = glucomannan, β-glucan, or HEC). CBM, carbohydrate-binding module; HEC, hydroxyethyl cellulose; BMCC, bacterial microcrystalline cellulose.

Initially, a pull-down assay was performed to determine whether DdCBM8 binds to insoluble polysaccharides. About 10 micrograms of DdCBM8 were incubated with 35 mg/ml of Avicel and bacterial microcrystalline cellulose (BMCC). After several washing steps, the protein bound to the polysaccharides was detected with SDS gel. As shown in Figure 2*A*, DdCBM8 was able to bind to both the polysaccharides (protein is present in the insoluble fraction). An attempt to determine the affinity constant to insoluble polysaccharides was performed using depletion isotherm assay, where different amounts of DdCBM8 were incubated with constant concentration of each ligand, and the plot of bound *versus* unbound (free) protein could reveal the affinity constants. In fact, the binding was confirmed (Fig. 3*A*). However, saturation was not achieved, precluding determination of the affinity of DdCBM8 for BMCC and Avicel.

The ability of DdCBM8 to bind soluble polysaccharides was assessed using an affinity gel electrophoresis (AGE). Basically, nondenaturing gels polymerized with different polysaccharides were used in electrophoresis, and the protein migration was reduced when binding occurred between protein and ligand, in comparison to a gel lacking the ligand (native). As shown in Figure 2*B*, DdCBM8 bound to xyloglucan, glucomannan, β-glucan, and hydroxyethyl cellulose (HEC), but not to xylan. Subsequently, intrinsic fluorescence spectroscopy was performed to monitor (Fig. 3*B*) and estimate the binding capacity of DdCBM8 to these polysaccharides (Fig. 3*C* and Table S1). The data were fit to a one-site binding model. The average molecular weight (MW) of polysaccharides was employed to determine the binding constants to these polysaccharides, although this approach may overestimate the affinity as each chain can often bind multiple CBMs. The DdCBM8 binding constant (*K*_*a*_) for glucomannan was 13.3 × 10^4^ M^−1^, whereas for β-glucan and HEC was 3.1 × 10^4^ M^−1^ and 0.66 × 10^4^ M^−1^, respectively. Affinity for xyloglucan was also detected; however, as no average MW was given by manufacturers owing to its heterogeneity, the binding affinity constant was not determined. Finally, no binding of DdCBM8 to xylan or any tested oligosaccharide, such as cellopentaose, cellohexaose, and xyloglucan heptasaccharide (X_3_Glc_4_ or XXXG, where X stands for a glucose decorated with xylose and G indicates an undecorated glucose) could be detected, based on fluorescence and isothermal titration calorimetry (data not shown) methods.

### General structural characteristics

Native DdCBM8 crystallographic structure was determined at 1.51 Å resolution. Initial phases were determined using the single-wavelength anomalous dispersion method using anomalous scattering of iodine, as described in the Experimental procedures section. At the end of refinement, the derivative dataset (resolution of 1.8 Å) displayed 13 iodine atoms with occupancies varying from 0.42 to 0.83. The statistics for data collection and refinement are presented in Table S2.

The final crystal structure presented a monomer in the asymmetric unit, and all amino acid residues (555–705) were built with well-defined electron densities. The amino terminal end also contained three residues (552–554; Ser, His, and Met) derived from the cloning vector, pET28a(+).

**Figure 4:**
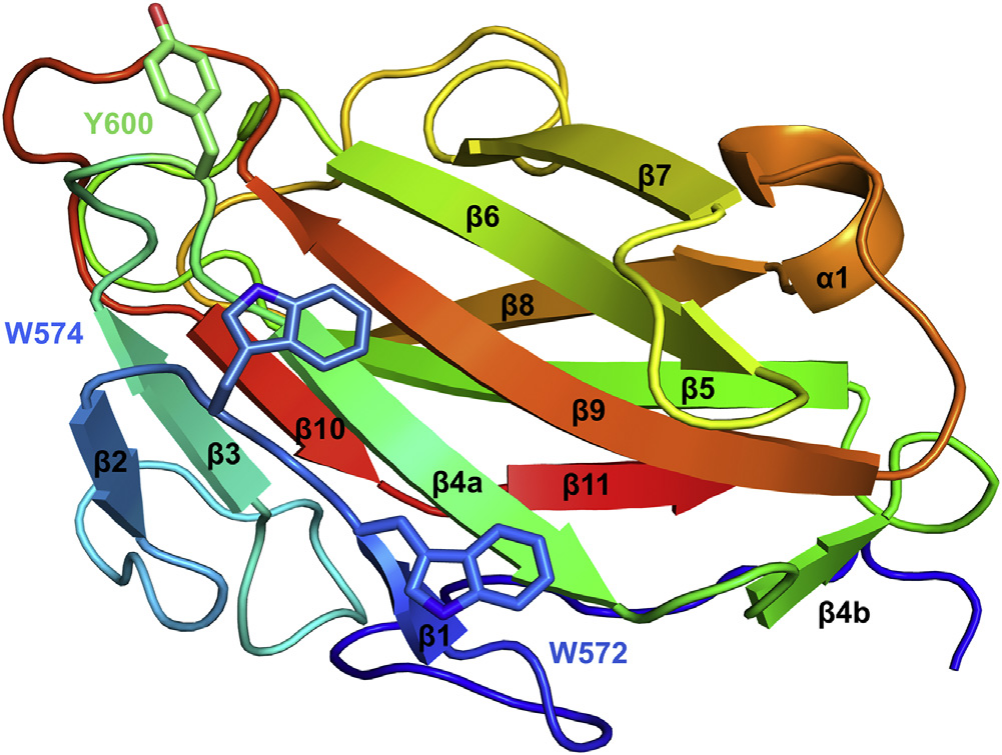
**Cartoon showing the 3-dimensional structure of DdCBM8.** The CBM has a typical β-sandwich fold, where two β-sheets, composed of 11 antiparallel β-strands, are packed against each other. The solvent-exposed aromatic residues involved in carbohydrate binding are depicted as *sticks* and labeled. CBM, carbohydrate-binding module.

DdCBM8 has a compact globular β-sandwich fold, typical of the CBMs, and composed of two antiparallel β-sheets with five and six β-strands (β1, β4, β6, β7, and β9, and strands β2, β3, β5, β8, β10, and β11) that are connected by loops and one α-helix (Fig. 4). A structural comparison conducted with DALI server (24) and PDBeFold server (25) revealed that DdCBM8 shared low similarity with members of other CBM families: CBM11 (Protein Data Bank [PDB] ID: 1V0A), with 2.02 Å rmsd; CBM30 (PDB ID: 1WZX), with 2.02 Å rmsd; and CBM29 (PDB ID: 1GWM), with 2.15 Å rmsd.

### Characteristics of the ligand-binding site

**Figure 5:**
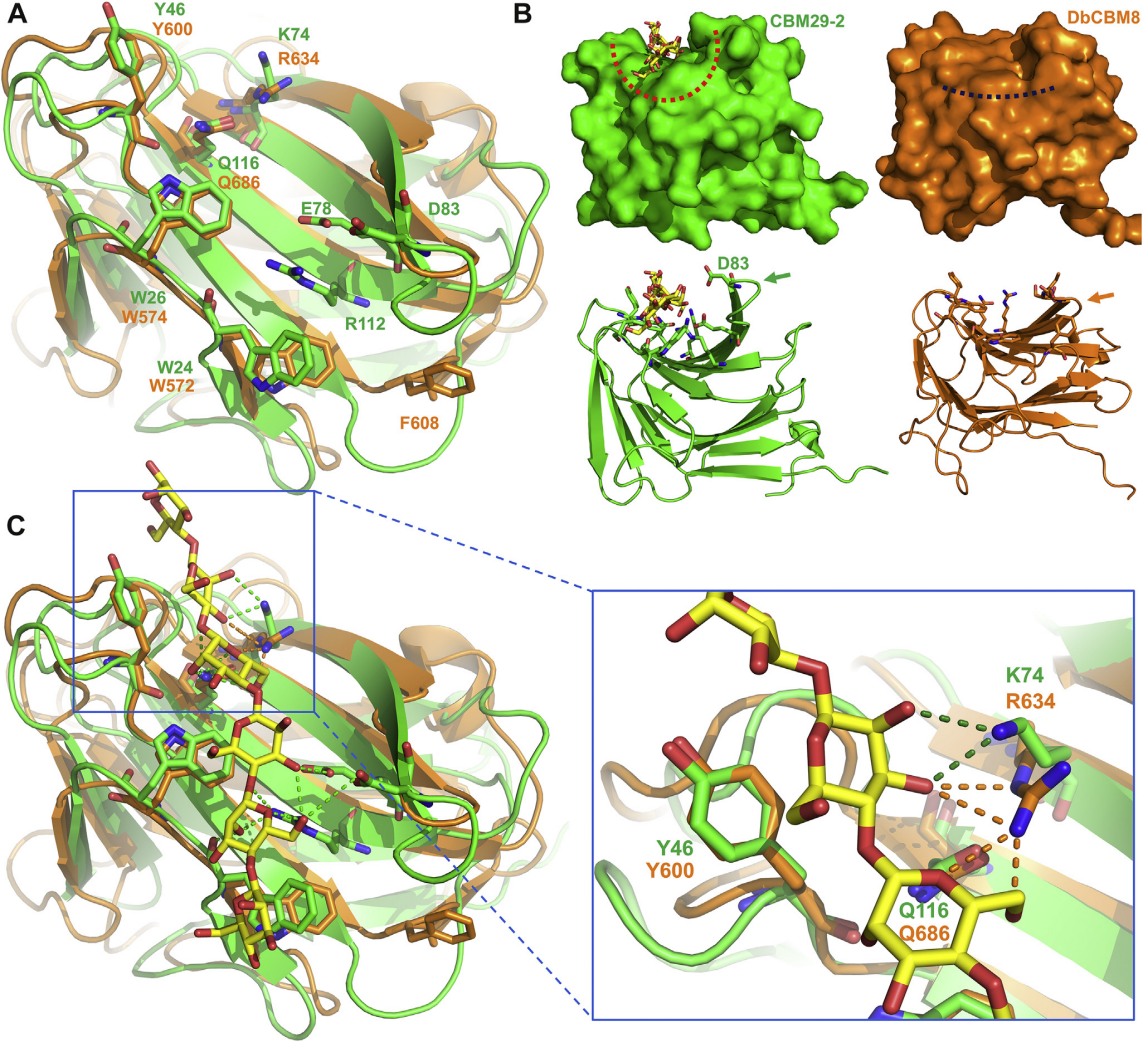
**Comparison between DbCBM8 (*orange*) and CBM29-2 (*green*; PDB:****1GWL****).***A*, superposition of both structures in *cartoon* representation, with ligand-binding site amino acids highlighted as *sticks*. Part of the binding sites (W572, W574, Y600, and Q686, in DdCBM8 numbering) has the same identity and very similar orientation, whereas other elements responsible for hydrogen bonding are absent in DdCBM8 (E78, D83, and R112, in CBM29-2 numbering). *B*, the binding site of CBM29-2 has a cleft shape (*red dashed line* in upper surface representation) to interact with ligands. However, the DdCBM8-binding site is flatter (*blue dashed line*), caused by a shorter loop and β-strand (*orange arrow*) where the amino acid D83 of CBM29-2 locates (*green arrow*). *C*, superposition of DdCBM08 (*green*) and CBM29-2 (*orange*) complexed with cellohexaose (PDB ID: 1GWL), evidencing the hydrogen bonds. CBM29-2 can form hydrogen bonds with the sugar hydroxyls *via* its K74 residue (*highlighted panel*). DdCBM08 has an arginine in the same position, R634, which theoretically could interact with ligand as well. However, the mutation of R634 into alanine did not change the mobility shift in AGE assay. AGE, affinity gel electrophoresis; CBM, carbohydrate-binding module; PDB, Protein Data Bank.

Attempts to cocrystallize and soaking with xyloglucan heptasaccharide (XXXG) and cello-oligosaccharides (cellopentaose and cellohexaose) failed, which is consistent with the absence of affinity determined for these ligands. However, three aromatic residues exposed to solvent (W572, W574, and Y600) formed a planar surface resembling a typical ligand-binding site in type A CBMs (Fig. 5). Despite the overall low structural similarity with other CBMs, these aromatic residues were significantly aligned with the binding site of a member of the CBM29 family, CBM29-2 (PDB ID: 1GWM) (26), which was cocrystallized with cellohexaose. Both CBMs (DdCBM8 and CBM29-2) present the same three aromatic residues in similar positions, which in the case of CBM29-2 were responsible for ligand binding through CH–π interactions. Furthermore, two other residues that are involved in ligand stabilization through hydrogen bonding in CBM29-2, share common positions: R634 and Q686 from DdCBM8 and K74 and Q116 from CBM29-2, respectively (Fig. 5*A*). The main difference in the binding sites is the presence of another three amino acids (E78, E83, and R112) in CBM29-2, which interact with the ligand through hydrogen bonds, but not in DdCBM8. Furthermore, DdCBM8 has three residues shorter β-strand (β7) compared with that in CBM29-2, which is responsible for creating a cleft where the ligand chain fits (Fig. 5*B*). The shorter β-strand found in DdCBM8 confers a planar shape for this region that probably allows crystalline polysaccharide binding.

### Contribution of specific amino acid residues to ligand binding

**Figure 6:**
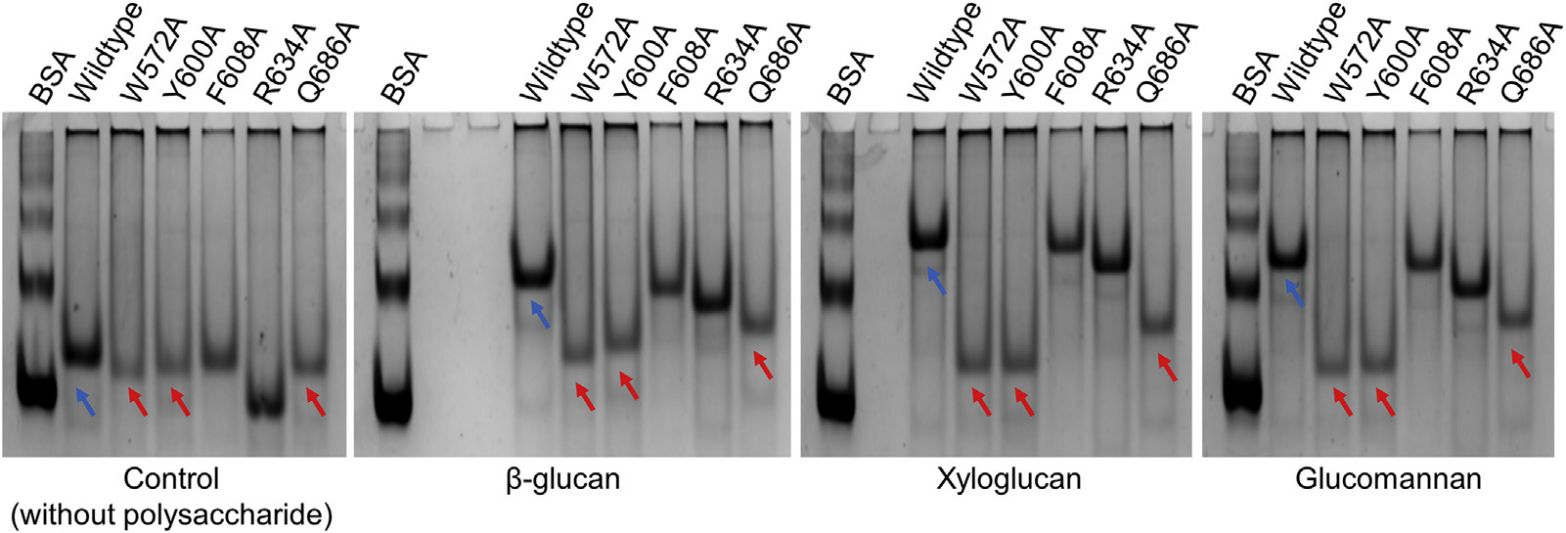
**Comparison of binding ability of mutant and wildtype (*blue arrows*) DdCBM8 to soluble polysaccharides.** Affinity gel electrophoresis assay experiment showed that mutants W572A, Y600A, and Q686 (*red arrows*) play an important role in ligand binding. The F608A and R634A mutations have no effect on binding. BSA was used as a control, as it does not interact with carbohydrates. BSA, bovine serum albumin; CBM, carbohydrate-binding module.

To confirm the ligand-binding site inferred from the crystallographic structure and to evaluate the role of each amino acid in the interaction, five residues of the sites were mutated to alanine (W572, Y600, F608, R634, and Q686), and the CBMs were then subjected to AGE assays using known ligands for DdCBM8 (Fig. 6). As predicted from the crystallographic structure, mutations in amino acids that form the core of the binding site through CH–π interactions (W572A and Y600A) abolished DdCBM8 from binding to all polysaccharides evaluated here. The same profile would be expected for the mutation of W574 but, even after several attempts, we were not able to generate the specific mutant W574A. No protein mobility shift was observed from mutations F608A and, R634A, indicating that F608 and R634 probably does not interact with the ligand. Although R634 seems to be in a proper position for hydrogen bonding with the ligand, as indicated by the superposed structures in Figure 5*C*, the mobility shift effect was negligible. Finally, Q686A led to an intermediate reduction in ligand binding, confirming its role on the binding site.

### Residues that comprise the ligand-binding site in DdCBM8 are conserved throughout the family

**Figure 7:**
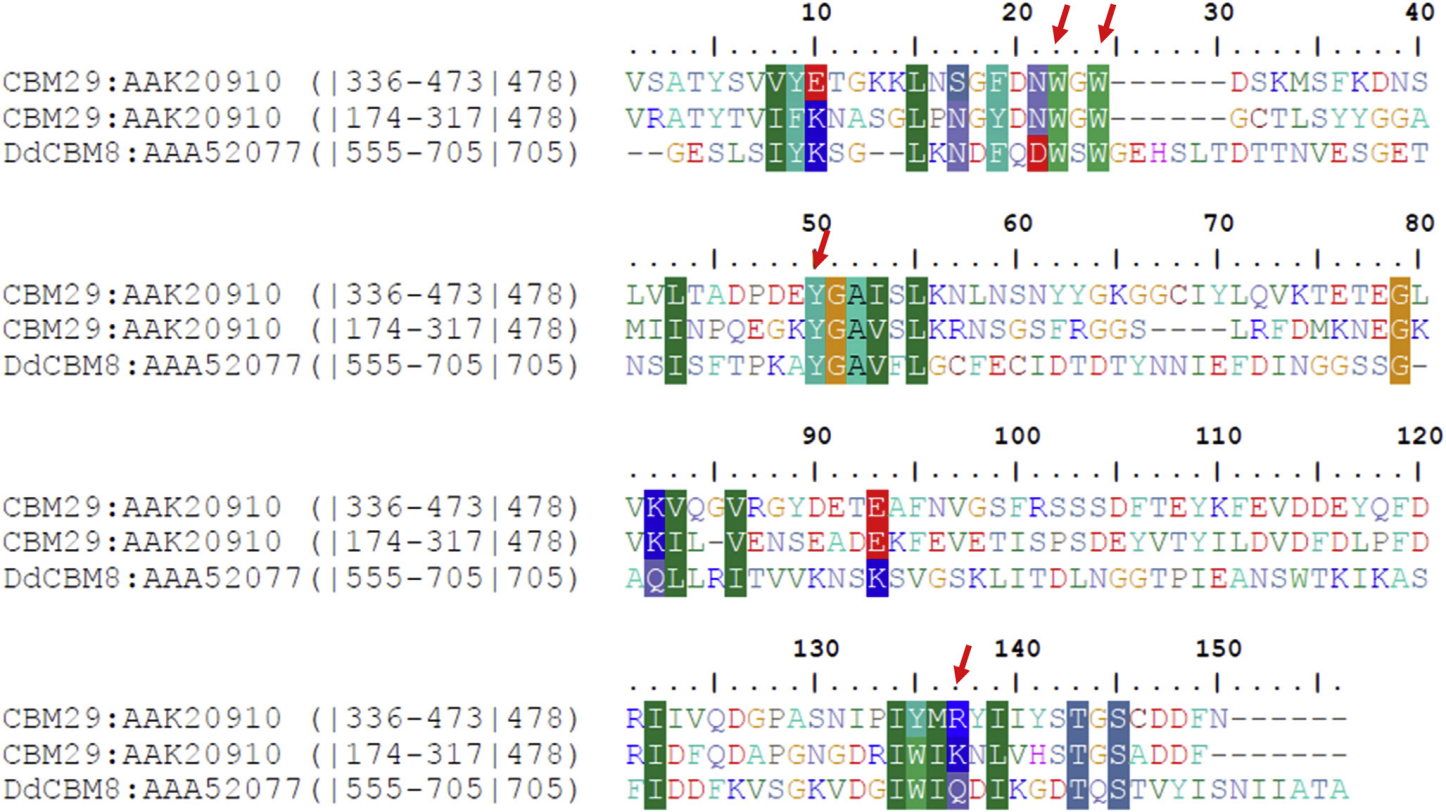
**Multiple alignment of DdCBM8 and two sequences from CBM29 family.** Database source and the corresponding accession numbers are shown before the alignment. Start and end positions of CBMs and residue numbers of full-length sequences are given in parentheses. Blocks of similar amino acids (using threshold at 70%) are shown on colors background. The amino acids that compose the binding sites are indicated by *red arrows*. CBM, carbohydrate-binding module.

The amino acid sequence of DdCBM8 was aligned with representative members of the CBM8 family (Fig. S1). In general, the sequences showed great variability. However, the predicted residues that compose the binding site (W572, W574, Y600, and Q686) were highly conserved. The residues F608 and R634, which according to our data did not influence protein–carbohydrate interaction, were not conserved among CBM8 family members. Besides the amino acid residues involved in binding and the other conserved positions found in the alignment are buried in the protein structure and do not seem to play any role in CBM function. A second alignment (Fig. 7) performed with DdCBM8 and the two members described in CBM29 family (26, 27) showed that the aromatic residues composing the binding site were conserved as expected. However, the CBM8 and CBM29 family members display low sequence identity and, therefore, it is difficult to infer any common ancestry.

## Discussion

The CBM8 family has 109 members reported to date, of which 99 belong to bacteria and the other two belong to *D. discoideum*, which is a slime mold Amoeba considered as a nonmammalian biomedical and pharmacological model owing to its cellular structure, intracellular signaling, and host–pathogen interaction similarities with mammalian cells (28, 29). The first and unique report of this family is related to the description of an endo-β-1,4-glucanase from *D. discoideum*, named CelA (23). Full-length CelA was able to bind to Avicel but not the cellulase domain itself or the cellulase with linker region (TETP repeat) (13). Thus, the C-terminal domain from CelA (described in this article as DdCBM8) was responsible for Avicel binding.

Following the previous report (23), the pull-down assay confirmed the affinity of DdCBM8 for Avicel and showed that it was able to bind to BMCC as well. Since BMCC has a significantly higher proportion of crystalline than amorphous cellulose (∼95%), as compared with Avicel (∼60%) (30), we can suppose that DdCBM8 has affinity for the crystalline portion indeed. However, the data indicate that this affinity is significantly lower than that observed for other type A CBMs (31, 32, 33). Subsequently, it was verified that DdCBM8 had a high affinity for soluble polysaccharides, especially glucomannan. The binding constant determined for glucomannan is similar (13.3 × 10^4^ M^−1^) to that found for type B CBMs (from 0.3 × 10^4^ to 5.8 × 10^4^ M^−1^) (26,34). Collectively, the results lead to an uncertainty in DdCBM8 classification as a type A or B.

The present study is the first structure of a CBM8 family reported to date. The crystallographic structure of DdCBM8 showed typical folding of the CBMs, but no similar structure was found in other families. Nevertheless, its binding site was inferred by structural comparison with members of the CBM29 family and further confirmed *via* mutagenesis of key amino acids. The planar binding site observed in the DdCBM8 structure would lead to its classification as a type A CBM. However, similar to CBM81, DdCBM8 is another exception to CBM-type canonical classification. It could be a type B with high affinity for soluble (single chain) polysaccharides or type A owing to the planar binding site of the protein. However, DdCBM8 lacks binding to oligosaccharides and has a low affinity for crystalline cellulose.

The unique characteristics of DdCBM8, together with its broad specificity, may contribute to a better understanding of CBM function and its future biotechnological applications. The structure of DdCBM8 has no parallel in other CBM families, which has a planar binding site that would lead to its classification as a type A CBM. However, DdCBM8 lacks binding to oligosaccharides and has a low affinity for crystalline cellulose, which are functional properties of type A CBM. On the other hand, DdCBM8 presents type B functional characteristics because of the high affinity for soluble (single chain) polysaccharides. Therefore, the structural characteristics and broad specificity of DdCBM8 will drive discussions and revision about this canonical classification and promote advances on the current understanding of CBM interaction and classification.

## Experimental procedures

### Protein expression and purification

The gene encoding the CBM DdCBM8, found in an endoglucanase from *D. discoideum* (UniProt ID: P22699), was synthesized by GenOne Biotechnologies from nucleotides 1663 to 2115 (amino acids 555–705). The commercial vector containing the gene was digested with NdeI and NotI restriction enzymes and cloned into the expression bacterial vector pET28a(+). Cloning was verified using DNA sequencing. The final construct encoded DdCBM8 fused to an N-terminal His tag with a site for thrombin protease cleavage for tag removal.

Recombinant DdCBM8 was expressed in *E. coli* strain BL21 (DE3) (Thermo Fisher Scientific). A single colony was used to inoculate 10 ml LB medium supplemented with kanamycin (50 μg/ml) as a starter culture. Then, LB medium (4 l) was cultured at 37 °C until absorbance of ≈0.6 at 600 nm, followed by induction with 0.5 mM IPTG for 16 h at 16 °C. Cells harvested by centrifugation were suspended in binding buffer (20 mM Tris–HCl, pH 8.0, 200 mM NaCl, 5 mM imidazole, and 20% glycerol) and incubated on ice with lysozyme (1 mg/ml) for 30 min. Cells were sonicated, and the clarified supernatants were incubated with TALON resin for 3 h at 25 °C. The beads were washed with 10 column volumes of washing buffer (20 mM Tris–HCl [pH 8.0], 200 mM NaCl, 10 mM imidazole, and 20% glycerol), and the retained proteins were eluted with wash buffer containing 250 mM imidazole. After His tag removal with thrombin, the protein was purified with a HiLoad 16/60 Superdex 75 prep grade column equilibrated with 20 mM Tris–HCl (pH 8.0), 150 mM NaCl, and 2% glycerol. Purified DdCBM8 was stored at 4 °C.

### Insoluble polysaccharide-binding assay

The pull-down assay was performed as described previously (35), with some modification. Ten micrograms of purified protein were incubated in 200 ml solution containing 35 mg/ml of Avicel or BMCC, dissolved in 25 mM ammonium acetate (pH 5.0), for 20 min at 20 °C and under 1000 rpm agitation. The mixture was centrifuged at 12,000*g* for 15 min, and the soluble fraction was collected, concentrated, and mixed with SDS sample buffer. The insoluble fraction was washed three times with 25 mM ammonium acetate (pH 5.0) and 1 M NaCl. After centrifugation, the pellet was resuspended in 100 ml SDS sample buffer. Soluble and insoluble fractions were analyzed using SDS-PAGE.

Ligand-binding quantification was measured using a depletion isotherm assay (31, 36). Reactions containing 1% ligand, 25 mM ammonium acetate (pH 5.0), and variable amounts of protein (4–155 μM) were incubated for 2 h at 20 °C and 1000 rpm. Samples were centrifuged at 12,000*g* at 20 °C for 5 min, and the protein concentration of the supernatant (unbound fraction) was measured using the Bradford method from Bio-Rad.

### AGE

AGE was performed as described previously (37, 38). Native polyacrylamide gels were prepared consisting of 8% acrylamide utilizing 0.2% of the following soluble polysaccharides: HEC, xylan, xyloglucan, glucomannan, and β-glucan. The gels were prepared using Tris-acetate buffer (pH 8.3) plus EDTA in the absence of SDS. 2-Mercaptoethanol was excluded from the loading buffer, and proteins were not heated at 95 °C prior to loading onto the gel. In all experiments, DdCBM8 was run simultaneously in the native gel with or without incorporated soluble polysaccharides. Bovine serum albumin was used as the control. Electrophoresis was carried out at 125 V at 18 °C for 2 h. Proteins were visualized by staining with Coomassie Brilliant Blue R-250.

### Intrinsic fluorescence spectroscopy

All fluorescence measurements were performed using a Cary Eclipse Fluorescence Spectrophotometer (Varian) using a 10 mm path-length quartz cuvette with DdCBM8 (2.0 μM) in 20 mM sodium phosphate (pH 7.4), 50 mM NaCl buffer, at 20 °C. The excitation wavelength was 290 nm, and the emission intensities were measured over the wavelength of 300 to 450 nm. Five scans were averaged for each experiment. The emission intensities were corrected for background fluorescence caused by buffer and carbohydrates, for dilution, and for inner filter effects, and all the experiments were done in triplicate. Quantitative binding experiments were performed by titration of the appropriate carbohydrate. The binding constant (*K*_*a*_) and the number of binding sites (n) were calculated using the following relationship:

$$\text{log}\frac{\left(F_{0}-F\right)}{F}=\text{log}K_{a}+n\text{log}\left[Q\right]$$

where *F*_0_ and *F* are the fluorescence intensities in the absence and presence of the ligand (*Q*), respectively, [*Q*] is the ligand concentration. In this case, *K*_*a*_ is determined by the linear coefficient, and *n* (angular coefficient) gives the number of sites. The MWs of the carbohydrates are an average value given by the manufacturers: glucomannan (MW = ∼250 kDa), β-glucan (MW = ∼179 kDa), and HEC (MW = ∼90 kDa).

### Crystallization and data collection

Pure DdCBM8 was concentrated to 9.7 mg/ml and subjected to initial crystallization screening with the aid of a Honey Bee 963 robot from the ROBOLAB facility at the Brazilian Biosciences National Laboratory. The drops containing 0.5 μl of protein solution and 0.5 μl of reservoir solution were placed in sitting-drop vapor-diffusion plates and incubated at 18 °C. Commercial kits (Hampton, Qiagen, and Rigaku) were used as the initial conditions. The first hits led to the refinement of the conditions using hanging-drop vapor-diffusion plates, where the drops contained 2 μl of protein solution and 2 μl of reservoir solution and were incubated at 18 °C.

The crystals of DdCBM8 were obtained in condition containing 1.6 M disodium dl-malate, soaked in cryoprotection solution (20% ethylene glycol and crystallization solution), and flash cooled in a stream of gaseous nitrogen at 100 K. For derivatization, a DdCBM8 crystal was soaked in cryoprotection solution containing 0.6 M sodium iodide. The X-ray diffraction data were collected at the MX-2 beamline of the Brazilian Synchrotron Light Laboratory (LNLS) using a PILATUS2M detector (Dectris).

Collected data were integrated with iMosflm (39) and XDS (40) and scaled with AIMLESS (41). Initial phases were determined using the single-wavelength anomalous diffraction method using the program AUTOSOL (42) from PHENIX (43). The phases from a native dataset were identified by molecular replacement using Phaser (44) with DdCBM8/I as the search model. The models were refined using PHENIX (43) and interspersed with manual adjustment in Coot (45). Final models were deposited in the PDB with codes 7T7Z and 7T7Y, respectively, to native and derivative data.

### Site-directed mutagenesis

Site-directed mutagenesis was carried out using the Quikchange kit (Agilent). The DdCBM8/pET28a vector was used as a template, and the primers carrying the mutations were as follows: W572A, 5′-CTTCCAGGATGCGAGCTGGGGC-3′ and 5′-TCGTTCTTCAGACCAGATTTG-3′; W574A, 5′-GGATTGGAGCGCGGGCGAGCAC-3′ and 5′-TGGAAGTCGTTCTTCAGAC-3′; Y600A, 5′-TCCAAAGGCCGCTGGTGCTGTG-3′ and 5′-GTGAAGGAAATAGAATTGG-3′; F608A, 5′-CCTGGGTTGCGCCGAATGCATTG-3′ and 5′-AACACAGCACCATAGGCC-3′; R634A, 5′-GCACAGCTGCTGGCTATCACTGTGG-3′ and 5′-GCCAGAGCTGCCACCG-3′; and Q686A, 5′-GGCATCTGGATTGCGGACATTAAGGG-3′ and 5′-GTCCACTTTGCCGGAT-3′. The mutant proteins were expressed and purified as described in the previous section.

### Sequence alignment of CBMs

Based on CBM8 and CBM29 families available in the dbCAN (46), the amino acid sequences of the corresponding domains were retrieved from GenBank and UniProt with a cutoff minimum E-value of 1e^−20^. Multiple sequence alignments were performed using ClustalX (47) and the matrix Blossum62. The alignment obtained was manually refined using Bioedit (48) based on the conserved amino acid residues.

### Carbohydrate sources

All soluble polysaccharides and oligosaccharides were purchased from Megazyme International, except for xylan (beechwood) and HEC, which were from Sigma. The bacterial cellulose membrane production has been described previously (22, 49).

## Data availability

The data that support the findings of this study are available from the corresponding author upon reasonable request.

## Supporting information

This article contains supporting information.

## Conflict of interest

The authors declare that they have no conflicts of interest with the contents of this article.
