# Supplementary material for: Unique properties of a Dictyostelium discoideum carbohydrate-binding module expand our understanding of CBM–ligand interactions
Source: J Biol Chem. 2022 Apr 1;298(5):101891. doi: 10.1016/j.jbc.2022.101891 (PMC9079177; doi:10.1016/j.jbc.2022.101891)
Supplement: Supplementary Figure S1 and Tables S1–S2 [file mmc1.docx]

**Supporting Information**

The unique properties of a carbohydrate-binding module family 8 from *Dictyostelium discoideum* deepen the understanding of CBM-ligand interactions.

Marcelo Vizona Liberato ^1a^, Bruna Medeia Campos ^2a^, Geizecler Tomazetto ^1^, Lucy Isobel Crouch ^3^, Wanius Garcia ^4^, Ana Carolina de Mattos Zeri ^5^, David Nichol Bolam ^3^, and Fabio Marcio Squina ^1^*

From the ^1^ Programa de Processos Tecnológicos e Ambientais, Universidade de Sorocaba (UNISO), Sorocaba, SP, Brasil; ^2^ Laboratório Nacional de Biociências (LNBio), Centro Nacional de Pesquisa em Energia e Materiais (CNPEM), Caixa Postal 6192, CEP 13083-970, Campinas, São Paulo, Brasil; ^3^ Institute for Cell and Molecular Biosciences, The Medical School, Newcastle University, Newcastle upon Tyne NE2 4HH, United Kingdom; ^4^ Centro de Ciências Naturais e Humanas, Universidade Federal do ABC (UFABC), Santo André, São Paulo, Brasil; ^5^ Laboratório Nacional de Luz Sincrotron (LNLS), Centro Nacional de Pesquisa em Energia e Materiais (CNPEM), Caixa Postal 6192, CEP 13083-970, Campinas, São Paulo, Brasil.

Running title: Structural and functional properties of a CBM 8 member

^a^ These authors contributed equally to this work

*Correspondence to: Fabio Marcio Squina, fabio.squina@gmail.com.

Keywords: Carbohydrate-binding protein, X-ray crystallography, substrate specificity, cellulose, biofuel

**Supplementary Figures**


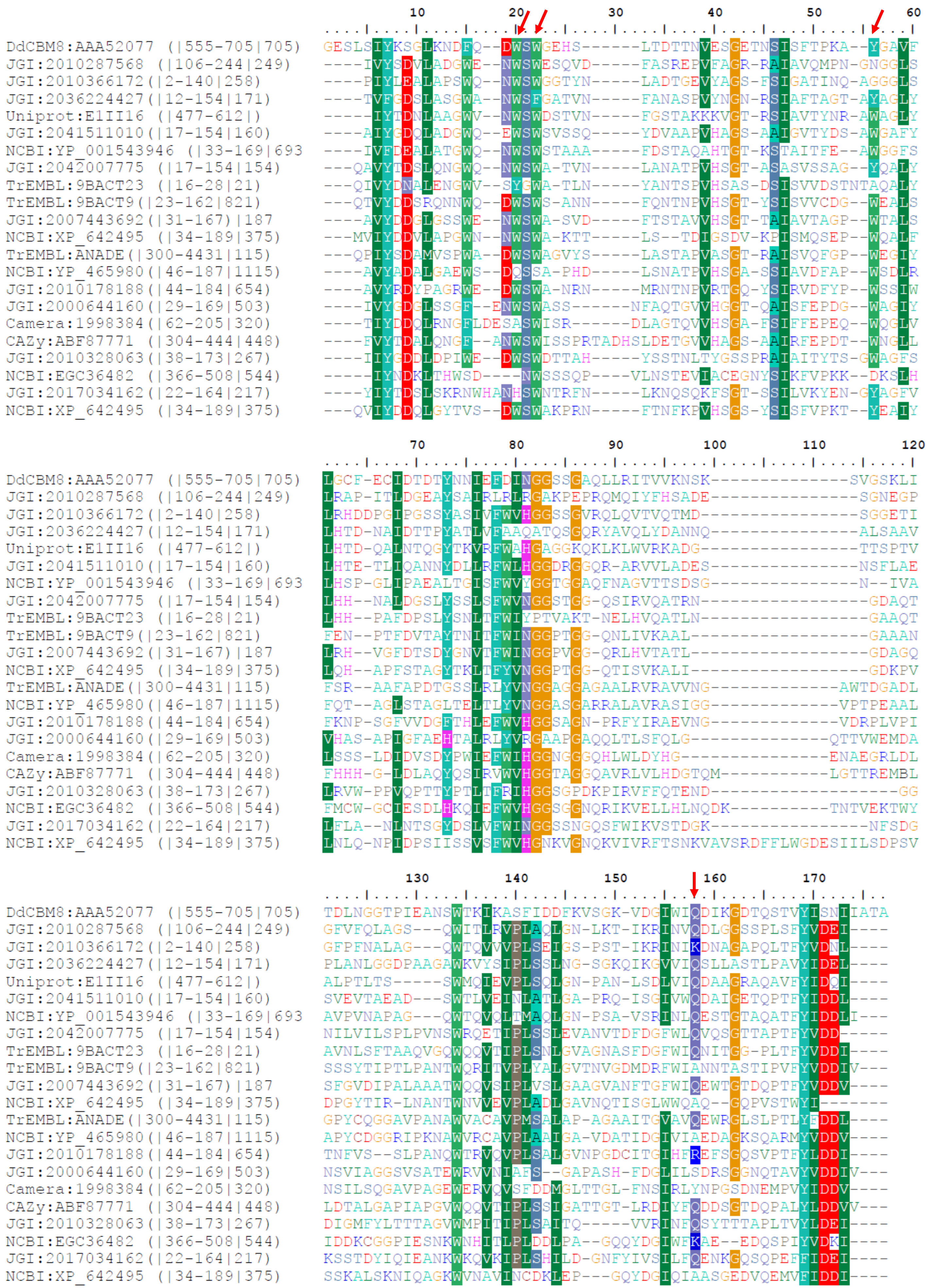


Figure S1. Multiple-alignment of DdCBM8 and representative members of CBM8 family. Database source and the corresponding accession numbers are shown before the aligned. Start and end position of CBMs and residues numbers of full-length sequences are given in parentheses. Blocks of similar amino acids (using threshold at 70 %) are shown on colors background. The key amino acids residues involved on protein-carbohydrate interaction in DdCBM8 are highlighted with arrows.

**Supplementary Tables**

Table S1. Binding constants (Ka), binding sites (n) and free energy changes (ΔG^0^) for polysaccharides binding into DdCBM8 determined by fluorescence quenching using a one-site binding model. The Gibbs free energy change (ΔG^0^) was calculated using the Van’t Hoff equation: ΔG^0^ = -RTln(K_a_), where T = 293 K is the absolute temperature, and R = 8.314 J/mol.K.

|  | **log(Ka)** | **n** | **r^2^** | **Ka (M^-1^)** | **ΔG^0^ (kJ/mol)** |
| --- | --- | --- | --- | --- | --- |
| **Glucomannan** | 5.12 ± 0.26 | 1.01 ± 0.04 | 0.98585 | (1.33 ± 0.07) x 10^5^ | - 28.74 |
| **β-glucan** | 4.49 ± 0.30 | 1.07 ± 0.05 | 0.98265 | (3.09 ± 0.21) x 10^4^ | - 25.19 |
| **HEC** | 3.82 ± 0.18 | 1.04 ± 0.04 | 0.98889 | (0.66 ± 0.04) x 10^4^ | - 21.43 |

Table S2 – Crystallographic data processing and refinement statistics for DdCBM8. Values in parentheses represent the highest resolution shell.

|  | native | I derivative |
| --- | --- | --- |
| **Data processing** |  |  |
| Diffraction source | LNLS Synchrotron | LNLS Synchrotron |
| Wavelength (Å) | 1.4586 | 1.8 |
| Space group | P22_1_2_1_ | P22_1_2_1_ |
| Cell dimensions |  |  |
| a, b, c (Å) | 36.74, 47.27, 79.45 | 36.79, 47.27, 79.34 |
| Resolution range (Å) | 47.27-1.46 (1.49-1.46) | 47.27-1.81 (1.85-1.81) |
| Total number of reflections | 215097 (3985) | 106102 (1909) |
| Number of unique reflections | 23773 (1049) | 12778 (605) |
| Completeness (%) | 96.9 (90.9) | 96.9 (78.9) |
| Redundancy | 9.0 (3.8) | 8.3 (3.2) |
| 〈 I/σ(I)〉 | 19.4 (4.8) | 12.0 (3.2) |
| R_pim_ | 0.023 (0.131) | 0.062 (0.314) |
| CC_(1/2)_ | 0.998 (0.956) | 0.973 (0.651) |
| **Refinement** |  |  |
| Rwork | 0.1523 | 0.2315 |
| Rfree | 0.1909 | 0.2475 |
| No. atoms | 1400 | 1246 |
| Protein | 1167 | 1183 |
| Ligands/ions | 14 | 7 |
| Water | 219 | 56 |
| Average B-factor (Å^2^) | 11.74 | 4.35 |
| Protein | 9.64 | 3.95 |
| Ligands | 11.49 | 10.03 |
| Water | 22.93 | 12.17 |
| R.m.s.d. |  |  |
| Bond (Å) | 0.004 | 0.012 |
| Angle (°) | 0.78 | 1.28 |
| Ramachandran |  |  |
| Favored (%) | 98.68 | 98.03 |
| Allowed (%) | 1.32 | 1.97 |
| Outliers (%) | 0.00 | 0.00 |
